# COVID-19 Health Crisis and Chronic Illness: Protocol for a Qualitative Study

**DOI:** 10.2196/28728

**Published:** 2021-09-09

**Authors:** Élise Ricadat, Aude Béliard, Marie Citrini, Yann Craus, Céline Gabarro, Marie-France Mamzer, Ana Marques, Thomas Sannié, Maria Teixeira, Marko Tocilovac, Livia Velpry, François Villa, Louise Virole, Céline Lefève

**Affiliations:** 1 Institut La Personne en Médecine Université de Paris Paris France; 2 Institut de la Recherche Saint Louis (IRSL) Université de Paris Paris France; 3 Centre de Recherche Psychanalyse, Médecine et Société (CRPMS) Université de Paris Paris France; 4 Centre de Recherche Médecine, Science, Santé, Santé mentale, Société (CERMES 3) Université de Paris Paris France; 5 Représentante des Usagers Assistance Publique - Hôpitaux de Paris Paris France; 6 Institut d'Histoire et de Philosophie des Sciences et des Techniques (IHPST) Université Paris 1 - Panthéon Sorbonne Paris France; 7 Institut des Humanités en Médecine (IHM) Université de Lausanne Lausanne Switzerland; 8 Laboratoire Épidémiologie Clinique et Evaluation Economique appliqué aux populations Vulnérables (ECEVE) Université de Paris Paris France; 9 Unité de Recherche Ethics, Research, Translation (ETREs) Centre de Recherche des Cordeliers Université de Paris Paris France; 10 Unité Fonctionnelle d’Ethique Médicale Hôpital Necker- Enfants malades Assistance Publique - Hôpitaux de Paris Paris France; 11 Etablissement Public de Santé Mentale Ville Evrard Neuilly-sur-Marne France; 12 Association Française des Hémophiles Paris France; 13 Université Paris 8 Saint-Denis France; 14 Laboratoire Sciences, Philosophie, Histoire (SPHERE) Université de Paris Paris France

**Keywords:** chronic illness, care, prevention, vulnerability, health democracy, COVID, qualitative study, COVID-19, pandemic, risk

## Abstract

**Background:**

The acute nature of the COVID-19 pandemic has put a strain on health resources that are usually dedicated to chronic illnesses. Resulting changes in care practices and networks have had major repercussions on the experience of people with chronic disorders.

**Objective:**

This paper presents the protocol of the Parcours, Associations, Réseau, Chronicité, Organisation, Usagers, Retour d’expérience, Soins (PARCOURS)-COVID study. The aim of this study is to evaluate the effects of reorganization of the health system on the usual care network of patients with chronic illness, which fosters and qualifies the quality and continuum of care provided. The first objective of this study is to document these patients’ experiences through transformations and adaptations of their network, both in the practical dimension (ie, daily life and care) and subjective dimension (ie, psychosocial experience of illness and relationship to the health system). The second objective of the study is to understand and acknowledge these reorganizations during the COVID-19 lockdown and postlockdown periods. The third objective is to produce better adapted recommendations for patients with chronic illness and value their experience for the management of future health crisis.

**Methods:**

The PARCOURS-COVID study is a qualitative and participatory research involving patient organizations as research partners and members of these organizations as part of the research team. Three group of chronic diseases have been selected regarding the specificities of the care network they mobilize: (1) cystic fibrosis and kidney disease, (2) hemophilia, and (3) mental health disorders. Four consecutive phases will be conducted, including (1) preparatory interviews with medical or associative actors of each pathology field; (2) in-depth individual interviews with patients of each pathology, analyzed using the qualitative method of thematic analysis; (3) results of both these phases will then be triangulated through interviews with members of each patient’s care ecosystem; and finally, (4) focus groups will be organized to discuss the results with research participants (ie, representatives of chronic disease associations; patients; and actors of the medical, psychosocial, and family care network) in a research-action framework.

**Results:**

The protocol study has undergone a peer review by the French National Research Agency’s scientific committee and has been approved by the research ethical committee of the University of Paris (registration number: IRB 00012020-59, June 28, 2020). The project received funding from August 2020 through April 2021. Expected results will be disseminated in 2021 and 2022.

**Conclusions:**

Our findings will better inform the stakes of the current health crisis on the management of patients with chronic illness and, more broadly, any future crisis for a population deemed to be at risk. They will also improve health democracy by supporting better transferability of knowledge between the scientific and citizen communities.

**International Registered Report Identifier (IRRID):**

DERR1-10.2196/28728

## Introduction

Since the 1970s and 1980s, the rise of chronic diseases has contributed to the creation and promotion of a paradigm, leading to a broader definition of “medicine” around the notion of “care,” developed in medicine as well as in the fields of social sciences and moral philosophy. In order to potentiate its effects in terms of patients’ quality of life, care needs to be deployed in a continuum of multiple relationships and practices combining medical, psychological, ethical, and social approaches [1-4].

The measures that resulted from the COVID-19 pandemic have led to prioritization of acute care, placing particular strain on the resources usually dedicated to the management of chronic pathologies. The COVID-19 outbreak in France has been characterized by a highly centralized reorganization of the health system as a response to the epidemic. This reorganization focused on taking care almost only of patients with COVID-19. In the media and across social networks, health professionals denounced these challenges, adding to the issue of infected patients’ triage [5].

The management of a chronic disease requires the daily intervention and cooperation of many different actors, which define an ecosystem of care. Such an ecosystem is based on a network of actors and institutions—medical or nonmedical, which combines diverse approaches and practices and requires constant collaborations and negotiations [6,7]. This ecosystem of care is determined by the pathology and the specificities of medical follow-ups required, but it is also highly dependent on the patient’s background and social environment, resources, and living conditions. It entails patients’ empowerment and active participation in their care support [8-11]. COVID-19 lockdown measures seem to have disrupted the ecosystem of care for patients with chronic illness on two levels. First, medical appointments were postponed or suspended, leading to self-medication practices without medical follow-up or storage of medicines essential to the management of the chronic condition [12]. The quality of life of patients with chronic illnesses was directly affected, as concerns rose about their health status and their risks to COVID-19 infection and as usual interactions with caregivers and health professionals were limited or modified, through the use of teleconsultation. In the case of mental health disorders, closure of psychiatric care facilities led to the termination of therapeutic activities, although these were regarded as essential for some patients who are very sensitive to the environment and social interactions. Second, care was reduced to chemical treatments, sometimes even intensified to compensate for the lack of psychosocial care, even though they cause side effects that increase the risk of COVID-19 infection [13]. It seems that prescribed care was doubly restricted—reduced to only vital surgical operations and deprived of several of its broader dimensions (ie, psychosocial care, pain control, and support).

Moreover, living with a chronic illness requires individuals to build their personal capacity to mobilize their existing knowledge based on experiences to collectively address medical, psychological, and social vulnerabilities. Individuals rely on the support of network of care to limit the effects of these vulnerabilities, yet the resulting quality of life rests on a delicate balance, which is constantly co-constructed and renewed. The experience of being autonomous or dependent does not come solely from the fact of having or not having support; it is rather a singular combination of material and human, family, and professional support [14]. The occurrence of a health crisis can deeply compromise this balance, which is already tangled in ordinary times, and thus increase the vulnerability of people living with a chronic disease. Conversely, research has shown that living with a chronic disorder also leads individuals to develop specific strategies and skills. Experiencing chronic diseases requires the mobilization and acquisition of important knowledge and resources, especially regarding the management of uncertainty and risk in the health field [15]. Participation of associative or targeted information networks of users and patients is required in structuring and disseminating this expertise related to the experience of chronic disease management [16-18]. This experience makes patients with chronic illness particularly sensitive to public health and solidarity issues. It is, therefore, likely that the individual or collective experience of chronic disease has not only been a factor of vulnerability but also a factor of resources, inventions, and adaptation in the current crisis.

For all these reasons, we hypothesized that the COVID-19 health crisis created an *unbalance* in the ecosystem of care and that the experiences of patients with chronic illness need to be better understood. We designed a research protocol to document the experiences of these patients and their caregivers. For this purpose, we organized a focus group with a panel of patients’ organization stakeholders, so as to identify their concerns and research needs. Several issues emerged from the focus groups. First, they acknowledged the inadequacy of COVID-19 public health measures with the experience of patients, as well as actors, professionals, or carers involved in the management of a chronic disease. Second, they asked for a better recognition of their experiential knowledge. Based on these elements, this paper presents the research protocol Parcours, Associations, Réseau, Chronicité, Organisation, Usagers, Retour d’expérience, Soins (PARCOURS)-COVID that uses a qualitative and participative methodology.

## Methods

### Objectives

The objective of the PARCOURS-COVID study is to document and highlight the experience of people with chronic diseases confronted to the health crisis by focusing on the changes that occur in their daily practices and ecosystem of care. It is led by the Institut de la Personne en Médecine, where social science researchers (history, philosophy and ethics, psychology, psychoanalysis, sociology, and anthropology), physicians, caregivers, and patient representatives collaborate to produce scientific knowledge in the medical humanities using a multidisciplinary approach developed over several years. The choice of adopting a qualitative approach by participatory research carried out with and among users, professionals, and caregivers, and supported by a strong involvement of chronic disease associations with respect to the project’s design and conduct, has three distinct goals, as outlined below:

#### Objective 1

The first objective is to value the lived experience, that is both psychological and social, as well as existential and practical, and their representations—the ways in which chronic illness, COVID-19, and the health crisis, in general, are thought about and made explicit in the discourse of individuals with chronic disorders. Semidirective individual interviews with patients will be conducted to that effect.

#### Objective 2

The second objective is to document changes in the practices and organization of the chronic care ecosystem in order to identify factors that are adaptive or, on the contrary, deleterious to maintaining its balance. Interviews with players in the medical, family, or caregiver network designated by patients will be conducted regarding to meet this objective.

#### Objective 3

The third objective is to generate and disseminate recommendations for a better adaptation of the health system for patients with chronic illness in the case of another health crisis, to ensure that the preparation and management this crisis will respect patients’ rights by promoting participation and involvement of patients and their associated organizations. Workshops with stakeholders in the health system will help transform and disseminate results to organizations and communities.

### Study Design and Participant Recruitment

The PARCOURS-COVID study received funding from the French National Agency of Research (Agence Nationale de la Recherche) for 9 months, from August 2020 to April 2021. The work schedule was developed in order to conciliate the project's feasibility requirements with the achievement of its scientific and operational objectives in a short time frame. Thus, we opted for a rapid qualitative approach and a participatory study design involving patients and associations’ members as research partners, an approached that has been previously validated [18]. This methodology is based on listening to the interviews, then synthetizing them according to themes predefined by the research team, while allowing new themes to emerge if necessary. Rapid qualitative methods are therefore partly deductive, while retaining their inductive component.

### Chronic Pathology Groups

Three groups of chronic pathologies were identified regarding the care networks’ specificities mobilized by them:

#### Group 1

Cystic fibrosis, a disease with a respiratory component and therefore a high risk of complication in the case of COVID-19, and kidney disease, both of which require a combination of hospital and nonhospital care (regular interventions by physiotherapists or home care nurses). Two main patients’ organizations (Renaloo and Overcoming Cystic Fibrosis—*Vaincre la mucoviscidose*) agreed to participate in the patient recruitment, as well as other stages of the research.

#### Group 2

Hemophilia, a condition that is most often self-managed by regular intravenous injections that are self-administered (2 or 3 days per week). The day-to-day management of patients with hemophilia essentially relies on a close relationship with health care professionals, mainly hospital doctors and nurses from rare disease expertise centers and, less frequently, general practitioners. The French Haemophilia Association agreed to assist in patient recruitment and to participate in the research process.

#### Group 3

Mental health disorders, a group of conditions that will help understand the specific impact of the health crisis for people with mental health disorders that are very sensitive to the environment and to social interactions, both of which were particularly disrupted during lockdown. Patients with mental health disorders require forms of care that are essentially based on relations, through consultations but also through day hospitals, peer groups, among other alternatives. A variety of professional partners agreed to assist in patient recruitment and participation in research. Previous collaborations have also been initiated with health care professionals in the psychiatric sector.

### Research Process

#### Overview

**Figure 1:**
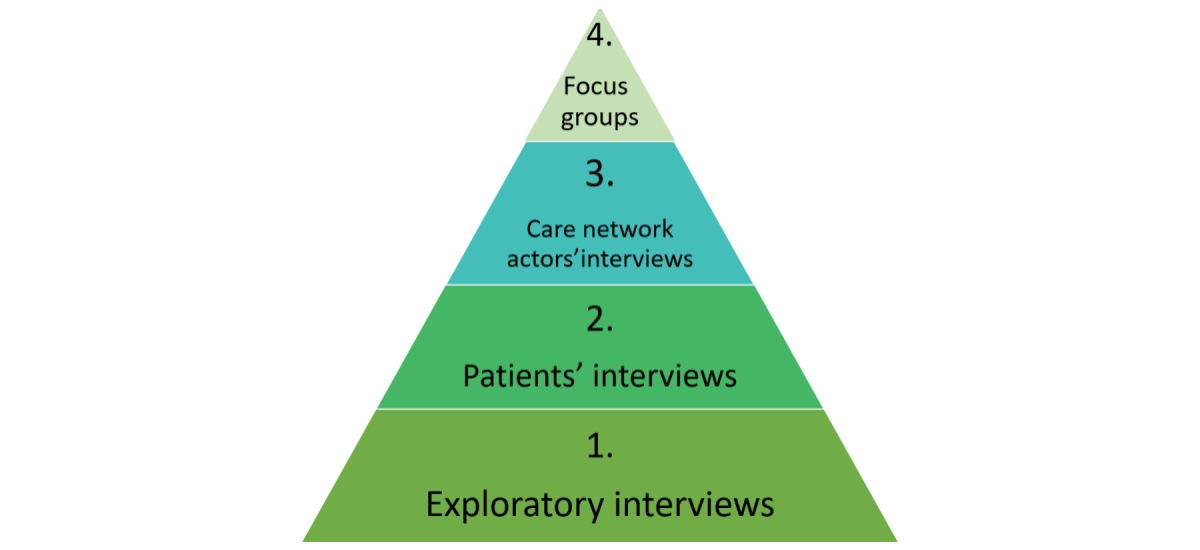
Four consecutive phases of the research study.

Four consecutive phases are scheduled, as follows: (1) preparatory interviews with medical or associative actors of each pathology’s field; (2) semidirective interviews with patients from the three abovementioned groups; (3) results from both phases will be triangulated through semidirective interviews with members of the patient’s care ecosystem, in order to review perspectives and gain a deeper understanding of the situation, through an analysis that will be carried out in close collaboration among social science researchers from several disciplines, patient associations, and caregivers; and finally, (4) focus-groups to discuss the results with research participants (Figure 1).

#### Phase 1: Exploratory Interviews

We will diversify entries in the field through our partnership with patient organizations. A series of preparatory interviews with key informants for each pathology will help identify specific situations and difficulties encountered during the crisis, including those concerning medical care, as well as the organization’s involvement. This phase will therefore include 16 interviews in all: with each organization leader and their health professionals, a doctor, and a front-line professional per pathology. One or two researchers from the team will carry out the interviews via phone or videoconferencing, or in person, if the situation allows it. We will also discuss with the partner organization and health professionals the pertinence of adding some pathology-specific criteria, such as type of treatments or access to care.

#### Phase 2: In-depth Patient Interviews

The second phase of the research will be based on interviews with individuals with chronic disorders, to collect data on their experiences during the health crisis. We will combine the recruitment through the partner organizations along with the recruitment of patients through the health professionals we met during phase 1. This will allow us to interview patients that are at located varying distances from patient organizations. A total of 7 to 10 adult patients per disease group, as identified above, will be interviewed. Patient recruitment in the first group will be equally divided between the two diseases. The participants’ situations will be diversified with regard to their socioeconomic characteristics and to ensure a balanced ratio of gender and various age groups and geographical locations (ie, between Paris and other regions or between the so-called COVID-19 “red and green zones”). This phase will thus include approximately 30 interviews with individuals with chronic disorders, conducted via phone by members of the research team (comprising only researchers or organization leaders that are part of the team).

#### Phase 3: In-depth Caregivers or Health Care Network Member Interviews

The third phase of the research will be based on interviews with members of the health care network of individuals with chronic disorders, in order to highlight their role as caregivers. They will be recruited with the cooperation and consent of patients from phase 2, who will be asked to name the two most important persons involved in the day-to-day management of their chronic disease. For instance, the care ecosystem could include health professionals, but also spouses, family, friends, or neighbors. At the rate of 1 to 2 actors identified per patient, the number of interviews can therefore be estimated to range between 30 and 60. The interviews will be conducted by members of the research team (only researchers or association leaders of the team).

#### Phase 4: Focus Groups for Feedback and Dissemination

In the fourth phase, focus groups will be organized to provide feedback to the participants and to discuss the results with them. Results from phases 2 and 3 will provide the basis for public policy recommendations. Participants of the focus group will include phase-2 and phase-3 interviewees on a voluntary basis. The focus groups will be led by the promoters of the present project, including the postdoctoral student (LV) who will be responsible for the organization and follow-up. Each focus group will discuss the main hypotheses and categories elaborated in by the research team and help deepen and validate them. The discussions will be recorded and transcribed and then linked to the empirical data gathered in phases 1 to 3. We will present the thematically analyzed interviews and the resulting hypotheses to focus group participants in order to discuss and validate them with the principal stakeholders. Data collected from these focus group will be analyzed to produce recommendations and outcomes. This participatory research process aims to improve their transferability to medical populations, citizens, and health authorities [19]. It will thus help raise awareness, as well as produce recommendations concerning the monitoring and care of patients with chronic disorders in the event of a health crisis, for different audiences: health authorities, scientific and professional networks, as well as service users and citizens. A specific website and a social network outreach strategy will be deployed for this phase.

### Data Analysis

Data analysis will take place continuously throughout the project and will begin in phase 1, with the production of summary sheets at the end of each interview. This process will help organize the data according to the various themes and perspectives that relate to the disciplines represented in the project. Emerging new themes will be included to feed conceptual categories. Return to the transcribed document will be possible, in order to find the exact verbatim of a statement identified in a sheet, to quote but also to identify the context. We will use the iterative process, which is characteristic of qualitative methodologies [20]. Our approach aims to build hypotheses through linking categories to empirical data.

During the descriptive phase of each case (ie, phases 2 and 3), a summary sheet will be produced for each interview, including a one-page summary of the interview from the interviewee’s point of view, as well as a brief paragraph on the interview’s relevance to the research issue. An account of the interviews and contacts made (ie, synthetic field diary) will also be produced by the researcher investigating the situation of each person within their health care network. The structure of these sheets will be discussed by all researchers involved in the study so that all the questions of interest to the various disciplines and user representatives can be considered. The results will be discussed and produced collectively through a series of working sessions in two formats, alternating between general meetings involving all research team members and smaller analysis workshops involving, each time, researchers of two disciplines and a member or an association or a professional.

By associating phases 2 and 3, we aim to cross-reference the points of view of different actors around the same situation and, thus, point to the role of some of them, which may remain hidden after a single interview [21]. This method will help reveal important but generally invisible players (pharmacist, medical secretary, etc), as well as adaptations of the forms of support between relatives that have been reconfigured by the containment measures. Comparing points of view also makes it possible to understand the plurality of definitions of the situation, an important dimension to be considered in the context of the health crisis, which is modifying everyone's expectations and requires continuous adaptation and negotiation. Finally, triangulating these two sources of data aims to capture the concrete and organizational reconfigurations of the health care ecosystems that are affected by the reorganization of health care resources, as well as their effects on the quality of medical and psychosocial care. Phases 2 and 3 will document more precisely objectives 1 and 2 defined above.

### Ethics Approval and Consent to Participate

Two separate interview guides have been co-constructed with the research team, based on the exploratory focus group: one is dedicated to “patient” interviews for phase 2, the other to “professionals, helpers, professionals” for phase 3. They are the result of a multidisciplinary approach, since researchers and representatives of patient associations suggested themes using their respective epistemological or experiential references.

The interviews will be conducted in compliance with health regulations via phone or videoconferencing. They will all be recorded based on oral consent of the individuals, fully transcribed and anonymized, and made available to all the members of the research team. The protection of the data collected will be specified in an information letter given to each person asked to participate in the research.

Having a consent form signed multiplies the documents with the identity of the persons. This is why we have not opted for this procedure, in favor of an oral recording of the consent before starting the interview which, in our opinion, better guarantees anonymity. This consent is subsequently recorded in writing in the transcript of the interview. Furthermore, having someone sign a consent form when working in Sciences Humaines et Sociales (SHS) can significantly alter the relationship between the respondent and the interviewer before starting the discussion. Moreover, in the context of the health crisis and protective measures that we are all currently subject to, we will not find ourselves physically come in face-to-face contact with the respondent. Getting the respondent to sign will be even more difficult and will therefore be done electronically.

Ethical approval was received from Comité d’Éthique de la Recherche (Research Ethics Committee) of the University of Paris. The protocol has been registered (institutional review board [IRB] no. 00012020-59; June 28, 2020).

### Availability of Data and Materials

Data will be stored in an encrypted form on a secure server of the University of Paris, in the cloud environment, accessible only to authorized researchers. The data processing implemented for the needs of this research will be done in compliance with the standard reference procedure (MR-004) and declared to the data protection officer’s registry at the University of Paris.

## Results

The protocol study has undergone a peer review by the French National Research Agency's scientific committee and has been approved by the Research Ethical Committee of the University of Paris (registration number: IRB 00012020-59 June 28th, 2020). The project received funding for the period August 2020 through April 2021. Expected results will be disseminated in 2021 and 2022.

## Discussion

As the exploratory phase of the research is being finalized, several operational issues emerged that foster discussion. The health crisis made it more complex to access the field, requiring adaptation from the research team. We encountered difficulties to access patients with mental illness. This situation was largely caused by a heightened variety of patients’ relationships with health care services, along with the fact that mental health organizations are loosely structured and scattered [22].

Realizing that the heterogeneity of chronic disease management requires a differentiated approach for each disease, we decided to adapt our recruitment to the specificities of each field. We changed our strategy to find other sources of entry into the field of mental health. We varied our entry points in the field, by diversifying the interlocutors: psychiatrists, psychologists, associations (the French National Union of Families and Friends of Mentally Ill People, UNAFAM), mutual self-help groups, patients’ homes (via the social media platform Clubhouse). We integrated a new specialized researcher into the research team, Ana Marques, who helped us enter into a psychiatric hospital in the department of Seine-Saint-Denis, where COVID-19 has had significant repercussions in terms of overloading structures, forcing hospitals to undergo a drastic reorganization [23]. These adjustments allowed us to diversify the profile of recruited patients, as we gained accessed to them through various care structures (eg, psychiatric hospitals, medical-psychological centers).

To conclude, the preparatory interviews were essential to inform the specificities of each field of chronic disease and allowed us to adapt our patient recruitment strategy to begin phase 2 of the research. At the end of our research, our findings will better inform the stakes of the current health crisis on the management of patients with chronic disorders and, more broadly, any future crisis for a population deemed to be at risk. They will improve health democracy by supporting a better transferability of knowledge between the scientific and citizen communities.

## Abbreviations

IRB: institutional review board
PARCOURS: Parcours, Associations, Réseau, Chronicité, Organisation, Usagers, Retour d’expérience, Soins
SHS: Sciences Humaines et Sociales
